# The *RPGRIP1*-deficient dog, a promising canine model for gene therapy

**Published:** 2009-02-18

**Authors:** Elsa Lhériteau, Lyse Libeau, Knut Stieger, Jack-Yves Deschamps, Alexandra Mendes-Madeira, Nathalie Provost, Francoise Lemoine, Cathryn Mellersh, N. Matthew Ellinwood, Yan Cherel, Philippe Moullier, Fabienne Rolling

**Affiliations:** 1 INSERM UMR 649, CHU-Hôtel Dieu, Nantes, France; 2 Service d’Urgences, Ecole Nationale Vétérinaire de Nantes, Nantes, France; 3 Clinique Vétérinaire Vetoceane, Vertou, France; 4 Animal Health Trust, Lanwades Park, Kentford, Newmarket, Suffolk, UK; 5 Department of Animal Science, Iowa State University, Ames, IA; 6 INRA UMR 703, Ecole Nationale Vétérinaire de Nantes, Nantes, France

## Abstract

**Purpose:**

To evaluate the *RPGRIP1*-deficient miniature longhaired dachshund (MLHD) dog as a potential candidate for gene therapy.

**Methods:**

Six *RPGRIP1*-deficient MLHD dogs from our dog colony have been observed for two years using a variety of noninvasive procedures. These included bilateral full-field electroretinograms (ERG) to evaluate retinal function, fundus photographs to evaluate retinal vascularization, and optical coherence tomographs (OCT) to evaluate retinal thickness. We also performed histological examination of hematoxylin- and eosin-stained retinal sections as well as sections labeled in situ by the terminal dUTP nick end labeling (TUNEL) method.

**Results:**

ERG findings showed that as early as 2 months of age, cone function was lost while rod function was preserved. However, by 9 months of age, both cone and rod functions could not be detected. Functional visual assessment based on the ability to avoid obstacles showed that vision was retained up to the age of 11 months. Both OCT and histopathology studies revealed a progressive thinning of the outer nuclear layer (ONL) over the first 2 years of age. TUNEL labeling identified apoptotic photoreceptor cell death as the cause of this thinning of the ONL.

**Conclusions:**

A treatment strategy should consist in initiating gene therapy as early as possible after birth to prevent or delay the loss of rod function. In the MLHD, successful subretinal delivery of a therapeutic vector is feasible at 2 months of age and may prevent or delay the loss of rod function.

## Introduction

Inherited retinal degenerations are a heterogeneous group of disorders that nearly always share one critical feature: degeneration of photoreceptors. Retinal dystrophies often lead to blindness. Currently, there are no effective treatments. To study the pathophysiology of retinal degeneration, animal models for which the genetic basis is understood are used. They share clinical features with their human couterparts and serve as valuable tools to aid in the development of therapeuties. For example, the close similarities between humans and dogs, in terms of the clinical characteristics of disease resulting from *retinal pigment epithelium-specific protein 65 kDa* (*RPE65)* gene mutations, make the *RPE65^−/−^* Briard dog a valuable model for the evaluation of gene therapy. Several groups, including ours, have now reported restoration of vision in *RPE65^−/−^* dogs using recombinant adeno associated virus (rAAV) mediated delivery of the human *RPE65* gene [1-3]. The success of the *RPE65^−/−^* dog gene therapy experiments was crucial in the development of human clinical trials to treat patients affected with Leber congenital amaurosis (LCA) or early onset retinal degeneration linked to a mutation in *RPE65* [4-6].

The mutation involved in a retinal degeneration previously described in miniature longhaired dachshunds has been identified [7,8]. This mutation consists of a 44 bp insertion in exon 2 of the *retinitis pigmentosa GTPase regulator-interacting protein 1* (*RPGRIP1*) gene, and introduces a premature stop codon [9].

Genetic defects in the *retinitis pigmentosa GTPase regulator* (*RPGR*) gene cause retinitis pigmentosa and defects in *RPGRIP1* cause LCA [10,11]. A study reporting a comprehensive mutational analysis of all known genes in 179 unrelated patients showed that *RPGRIP1* accounts for 4.5% of the cases [12].

Patients with *RPGRIP1* mutations have degeneration of both rod and cone photoreceptor cells, and in early life they experience a severe loss of central acuity, which leads to nystagmus. The functions of *RPGR* and *RPGRIP1* are not fully understood. Both *RPGR* and *RPGRIP1* localize in the photoreceptor connecting cilium, a thin bridge linking the cell body and the light-sensing outer segment. A study of *RPGRIP1^−/−^* mice demonstrated that *RPGRIP1* is a stable polymer in the connecting cilium where it tethers *RPGR* and that *RPGR* depends on *RPGRIP1* for subcellular localization and normal function [13]. This same study also suggested that *RPGRIP1* is required for disk morphogenesis, putatively by regulating actin cytoskeleton dynamics.

The objective of this study was to evaluate this LCA canine model as a potential candidate for gene therapy. For this purpose, we further characterized the kinetics of the retinal degeneration and the disease phenotype in the *RPGRIP1* deficient miniature longhaired dachshund (MLHD).

## Methods

### *RPGRIP1*-deficient dogs

A colony of MLHD dogs carrying the *RPGRIP1* mutation (a 44 nucleotide insertion that introduces a premature stop codon) was developed at the Boisbonne center (Ecole Nationale Vétérinaire de Nantes, Nantes, France). All animals were cared for in accordance with the Association for Research in Vision and Ophthalmology (ARVO) statement for the use of animals in ophthalmic and vision research. Dogs were housed in purpose-built, environmentally enriched facility with 12 h light-12 h dark cycle, they were fed with commercial diets and provided with water ad lib. Affected dogs A1-A4 were monitored from birth to 2 years of age. A1 died at the age of 11 months from an intestinal obstruction. Dogs A5 (a 10-year-old affected dog) and A6 (a 2-month-old affected dog) were sacrificed for histology study (Table 1).

**Table 1:** List of dogs and examinations performed.

**Dogs**	**ERG**	**Fundus photography**	**OCT**	**Behavioral study (once a month)**	**H&E**	**IF**	**TUNEL**
A1	2, 4, 9 m	2, 9 m	nd	2–10 m	11 m	nd	11 m
A2	2, 4, 9, 12 m	2, 12 m	5, 12 m	2–24 m	28 m	nd	28 m
A3	2, 4, 9, 12 m	2, 12 m	nd	2–24 m	nd	nd	nd
A4	2, 4, 9, 12 m	2, 12 m	nd	2–24 m	nd	nd	nd
A5	nd	nd	10 y	nd	10 y	nd	nd
A6	nd	nd	nd	nd	2 m	2 m	2 m
NA1	6 m	nd	nd	nd	nd	nd	nd
NA2	nd	nd	nd	nd	2 m	2 m	2 m

Abbreviations: affected dog (A); nonaffected dog (NA); electroretinography (ERG); OCT is optical coherence tomography; hematoxylin- and eosin-stained sections (H&E); immunofluorescence stained sections (IF); sections labeled in situ by the terminal dUTP nick end labeling method (TUNEL); months (m); years (y); not done (nd).

### Electroretinography

Dogs were dark-adapted for 20 min, and electroretinograms (ERGs) were performed under general anesthesia. Dogs were anesthetized with a combination of 40 µg/kg medetomidine (Domitor^®^; Pfizer, Paris, France) with 5 mg/kg ketamine (Imalgène^®^; Rhone Merieux, Toulouse, France), mixed in one syringe and given intravenously in a single dose. After endotracheal intubation, anesthesia was maintained with 1.5% isofluorane. Dogs were monitored by clinical observations, respiratory rate measurements, temperature measurements, oxymetry, and capnography. Rod and cone function was tested using simultaneous bilateral flash photopic and scotopic electroretinography. ERGs were recorded in a standardized fashion, according to the International Society for Clinical Electrophysiology of Vision (ISCEV) [14], using a computer-based system (Neuropack μ™ MEB-9102K, Nikon-Kohden, Tokyo, Japan) and contact lens electrodes (ERGjet®; Microponent, Le Cret-du-Locie, Switzerland).

In fully dark-adapted dogs, attenuated white flash (0.028 cd.s.m^−2^) was used to elicit a rod ERG b-wave. Four flashes, presented at a rate of 0.5 Hz, were used to average the rod system response.

To elicit a maximal mixed rod and cone response, we used a single white light flash (2.8 cd.s. m^−2^) . The maximal mixed rod and cone responses were averaged by using three flashes, presented at a rate of 0.1 Hz. Following a 10 min light adaptation, cone function was tested with 1 Hz white light flashes, and the cone flicker was tested with 30 Hz white light flashes, both in addition to a continuous rod desensitizing white background light. Ten to 20 responses were averaged for light-adapted recordings.

The *b*-wave amplitude was measured from the *a*-wave peak to the *b*-wave peak. Flicker amplitude was measured from the trough to the peak. Measurement method followed the recommendations in the ISCEV Standard for Clinical electroretinography.

### Behavioral studies

Using a camcorder, we recorded the visual behavior of affected dogs walking through an obstacle course in normal light. The number of collisions incurred by each dog as it went through the course was compared at different time points after birth (every month over a 2 year period).

### Fundus photography

The pupils of the dogs were dilated 20 min before anesthesia using tropicamide (Ciba Vision Faure^®^; Novartis, Annonay, France) and phenylephrine hydrochloride (10% Neosynephrine^®^; Novartis). Dogs were anesthetized with a combination of 40 µg/kg medetomidine (Domitor^®^, Pfizer, Paris, France) with 5 mg/kg ketamine (Imalgène^®^), mixed in one syringe and given intravenously in a single dose. Maintenance with isofluorane was not needed. After endotracheal intubation, dogs were clinically observed and their respiratory rates were monitored. Fundi were imaged using a Canon UVI retinal camera connected to a digital imaging system (Lhedioph win software; Lheritier SA, Saint-Ouen l’Aumône, France).

### Optical coherence tomography

Retinal morphology was assessed by optical coherence tomography (OCT; Stratus 3000; Zeiss, Jena, Germany). Dilatation of the pupils and intravenous anesthesia of the animals were performed as described in the previous section. At different time points, a 3 mm horizontal line scan was performed in the area located above the optical nerve head.

### Histology

Eyecups were obtained from normal and affected dogs and were fixed for 48 h in Bouin’s solution (Laurylab, Saint Fons, France). Tissue sections (5–7 µm thick) were cut from paraffin-embedded blocks on a microtome. For histopathological examinations, sections were stained using a standard hematoxylin and eosin staining protocol. H&S sections were viewed with bright-light microscopy (Nikon, Champigny sur Marne, France).

For immunofluorescence evaluation, paraffin-embedded sections were deparaffinized and rehydrated with phosphate buffered saline (PBS, 0.9 mM CaCl_2_, 0.49 mM MgSO_4_, 2.68 mM KCl, 1.47 mM KH_2_PO_4_, 136.89 mM NaCl, 8.10 mM NaH_2_PO_4_). The sections were blocked with 5% BSA in 0.2% Tween /PBS for 30 min at room temperature, washed at room temperature in 0.2% Tween/PBS with 1% BSA. Sections were then incubated overnight at 4 °C with 1:100 mouse monoclonal anti-Rho4D2 antibody [15] (kindly provided by Dr. Molday, University of British Columbia, Vancouver, Canada) or 1:1,000 peanut lectin agglutinin-conjugated with fluorescein isothiocyanate (PNA-FITC; Vector Laboratories Inc., Burlingame, CA) diluted in 0.2% Tween /PBS. Sections were washed with 1% BSA in 0.2% Tween/PBS at room temperature and labeled with anti-Rho4D2 antibody. They were then incubated with 1:400 FITC-conjugated donkey anti-mouse secondary antibody (Jackson ImmunoResearch Laboratories Inc., Newmarket, England) diluted in 0.2% Tween/PBS for 1 h 30 min at room temperature. Sections were then washed with 1% BSA in 0.2% PBS/Tween.

### TUNEL assay

To detect apoptosis, we employed the TUNEL technique, using a commercially available kit (In situ Cell Death Detection Kit, Fluorescein; Roche, Mannheim, Germany). TUNEL staining was performed according to the manufacturer's instructions and as described by Labat-Moleur [16]. A positive control was used. This was obtained by inducing DNA fragmentation with DNaseI treatment before TUNEL staining, which mimics the DNA fragmentation associated with apoptosis. The negative control consisted of performing the TUNEL labeling without including the TdT enzyme.

### Statistical analysis

Retinal thickness values were obtained with OCT on both eyes from dogs A1, A2, A3, and A4. At each time point (2, 6, 9, 12, 15, 18, 21, and 28 months) the thickness of the retinas was expressed as mean (µm)±1 SD. Comparisons of retinal thickness were performed by Student's *t*-test. A p<0.05 was accepted as significant. The Student test required two assumptions are met: 1) the samples must be distributed as a Gaussian distribution, which was a reasonable assumption; 2) the variances of the two samples must be equal. The second assumption was checked using a Fisher Test.

For time points 2, 6, and 9 months, the number of measurements was n=8, and included measurements taken from dogs A1, A2, A3, and A4. For time points 12, 15, 18, 21, and 28 months, the number of measurements was n=6, and included dogs A2, A3, and A4 (A1 died at the age 11 months).

## Results

### Kinetics of retinal function loss in *RPGRIP1*-deficient dogs

**Figure 1:**
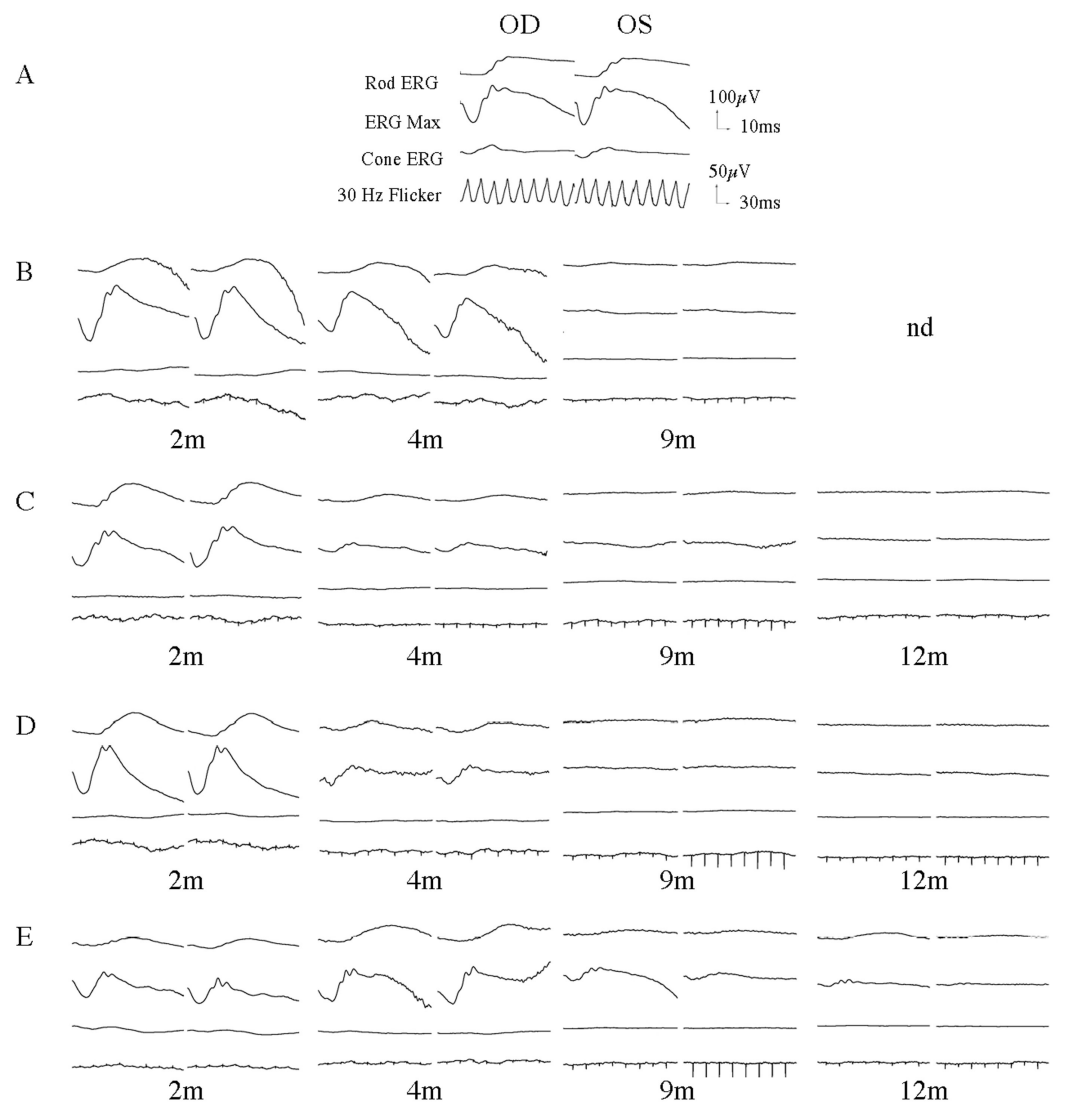
Bilateral full-field electroretinographic recordings of *RPGRIP1*-deficient dogs. **A:** Heterozygous carrier NA1 at the age of 6 months. **B:** Affected dog A1 at the age of 2, 4, and 9 months. **C:** Affected dog A2 at the age of 2, 4, 9, and 12 months. **D:** Affected dog A3 at the age of 2, 4, 9, and 12 months. **E:** Affected dog A4 at the age of 2, 4, 9, and 12 months. The top two recordings are low and high intensity scotopic responses. The bottom two recordings show photopic responses to light-adapted single flash and 30 Hz flicker stimuli.

Retinal function was tested using simultaneous bilateral full-field flash ERG (Figure 1). For all affected MLHD, the cone function (cone ERG and 30 Hz flicker) were barely detectable as early as 2 months of age. At this same time point, the rod function was similar between heterozygous carrier and affected animals, but progressively declined thereafter. At 4 months of age, the rod function was dramatically reduced for A2, A3, and A4 while A1 still displayed respectable signals. The ERG responses became undetectable at the age of 9 months for A1, A2, A3, and by 14 months for A4. It is interesting to note the substantial variation within the kinetic of retinal function loss from animal to animal. However, for all affected dogs, no retinal function could be detected by 14 months.

### Kinetics of vision loss in *RPGRIP1*-deficient dogs

We evaluated vision in all 4 affected MLHD using behavioral testing at different time points. This evaluation started at 2 months of age and was repeated every month over a 2 year period. Visual assessment was based on the ability to avoid obstacles in normal light. The obstacle course was arranged such that the placement of obstacles was unique from trial to trial. All dogs consistently avoided obstacles at the age of 9 months. At 11 months, A1, A2 and A3 failed to avoid obstacles, indicating they had loss of vision (a movie of A2 in the obstacle course can be viewed in Appendix 1). For A4, loss of vision was observed at 15 months. For all affected dogs, that residual vision was still detected during the 1–2 months following a complete loss of a measurable ERG signal.

### Thinning of retinal blood vessel and decrease of retinal thickness

**Figure 2:**
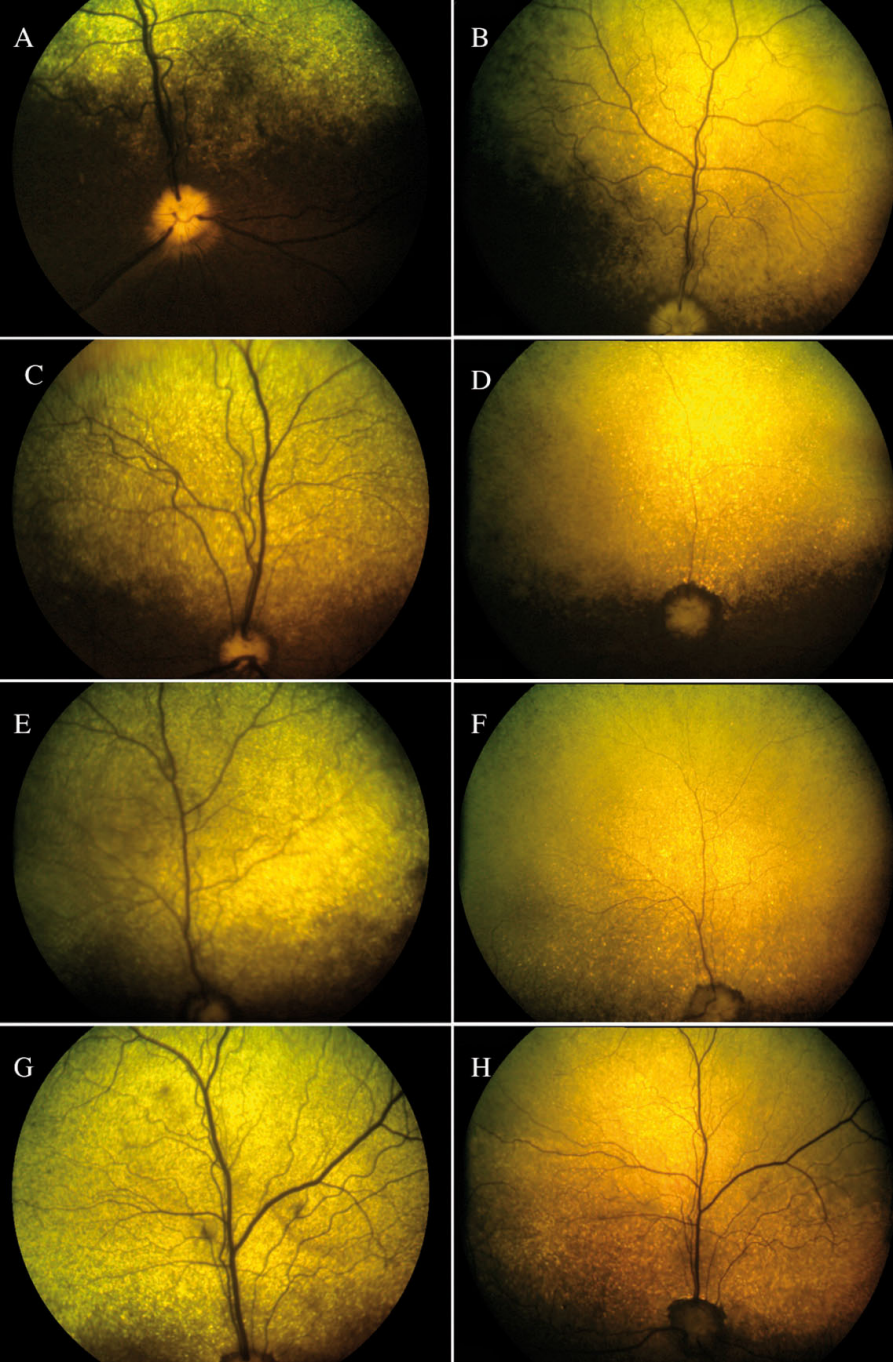
Fundus photographs in *RPGRIP1*-deficient dogs. **A, B:** A1 at the age of 2 and 9 months. **C, D:** A2 at the age of 2 and 12 months. **E, F:** A3 at the age of 2 and 12 months. **G, H:** A4 at the age of 2 and 12 months. There is progressive thinning of the retinal vasculature and an increase in hyperreflectivity of the tapetal area of the fundus.

Retinal morphology was monitored by color fundus photographs and by OCT in A2 to A4 for up to 2 years and up to 10 months for A1. For each affected MLHD, color fundus photographs displayed a progressive and dramatic thinning of the retinal vascularization, accompanied by a hyperreflectivity of the tapetal area of the fundus, over a 12 month period (Figure 2).

**Figure 3:**
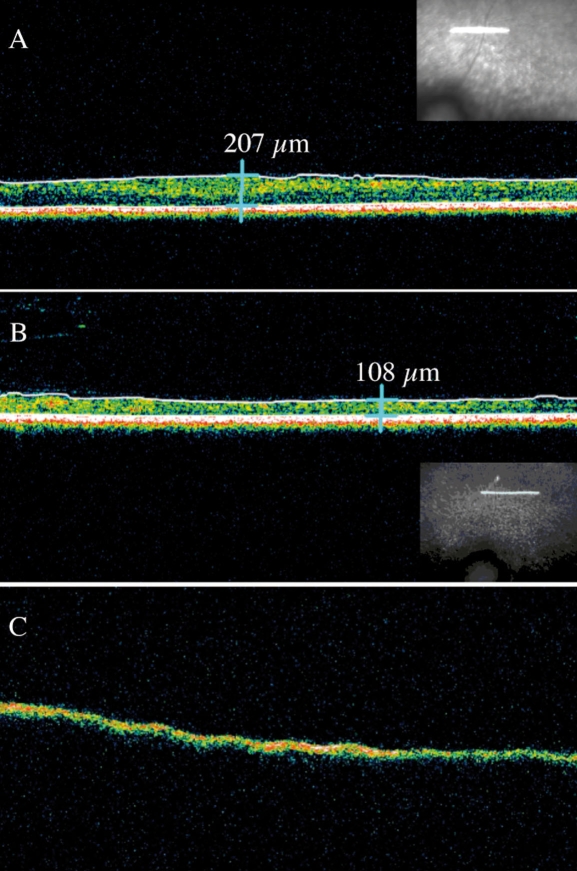
Images of OCT recordings of *RPGRIP1*-deficient dogs. The insets show the localization of the 3mm horizontal line scan above the optical nerve head. Notable decrease of the retinal thickness is observed in the affected dog A2 between 5 (**A**) and 12 months (**B**). Surprisingly, extreme thinning is seen in the 10-year-old affected dog A5 (**C**).

**Figure 4:**
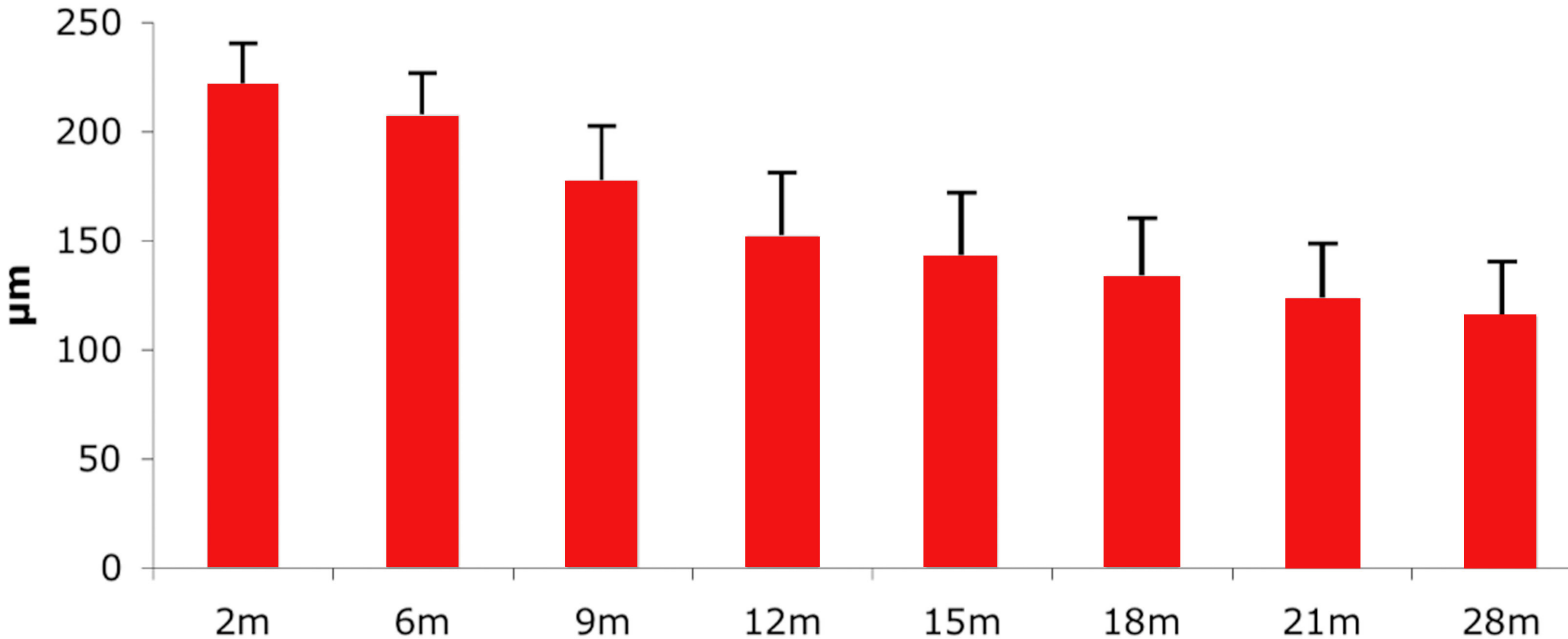
Decrease of full retinal thickness over time as observed on OCT scans. Bars (+1 standard deviation) represent retinal thickness measured on retinal sections from dogs of different ages. The Student *t*-test was performed to compare the mean retinal thickness at different stages of the disease. A p<0.05 was considered significant. The retinal thickness decrease was significant between 2 months and 28 months (p=8.7E07).

OCT is a non-contact, optical imaging technique that measures the intensity of backscattered light [17,18]. It produces a cross-sectional image, analogous to B-scan ultrasonography, but based on the optical reflectivity of the tissue, and thus, by using light instead of sound waves, provides an image with greater resolution. OCT imaging was performed in all affected animals at different time points. Long-term OCT monitoring in A1-A4 documented an important decrease of the retinal thickness between 5 and 12 months (Figure 3A,B). This decrease was statistically significant over the first 2 years of life (Figure 4). Interestingly, OCT imaging of A5, an older *RPGRIP1*-deficient dog that was monitored at the age of 10 years, displayed the RPE layer but no neuroretina, suggesting a complete degeneration of neuroretinal cells (Figure 3C). Moreover, dogs A2-A4 displayed lens opacities at the age of 21 months. Lens photographs of A5 at the age of 10 years showed a total cataract (data not shown).

### Histopathology and apoptosis of photoreceptors

**Figure 5:**
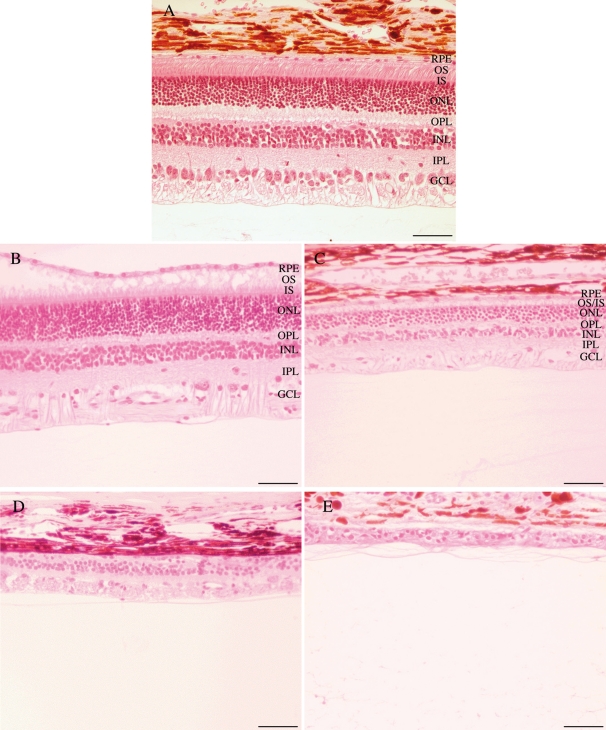
Histological sections of *RPGRIP1*-deficient dog’s retinas. **A:** Normal dog NA2 at the age of 2 months. **B:** Affected dog A6 at the age of 2 months. **C:** Affected dog A1 at the age of 11 months. **D:** Affected dog A2 at the age of 28 months. **E:** Affected dog A5 at the age of 10 years. These results confirm the thinning of the retina observed on OCT images. Abbreviations: retinal pigment epithelium (RPE); outer segments (OS); inner segments (IS); outer nuclear layer (ONL); outer plexiform layer (OPL); inner nuclear layer (INL); inner plexiform layer (IPL); ganglion cell layer (GCL). The scale bar represents 100 µm.

To better evaluate the retinal alterations observed on the OCT images, we sacrificed A6 at the age of 2 months, A1 at the age of 11 months, A2 at the age of 28 months, and A5 at the age of 10 years. The eyes were used for histopathological examination **(**Figure 5**).** Examination of hematoxylin- and eosin-stained sections revealed thinning of the outer nuclear layer between the age of 2 and 28 months, reflecting the progressive loss of photoreceptor cells. As seen in dog A5, by the age of 10 years, and as observed by OCT, a thin gliotic retina remained visible on histologic section with a complete lack of the photoreceptor layer.

**Figure 6:**
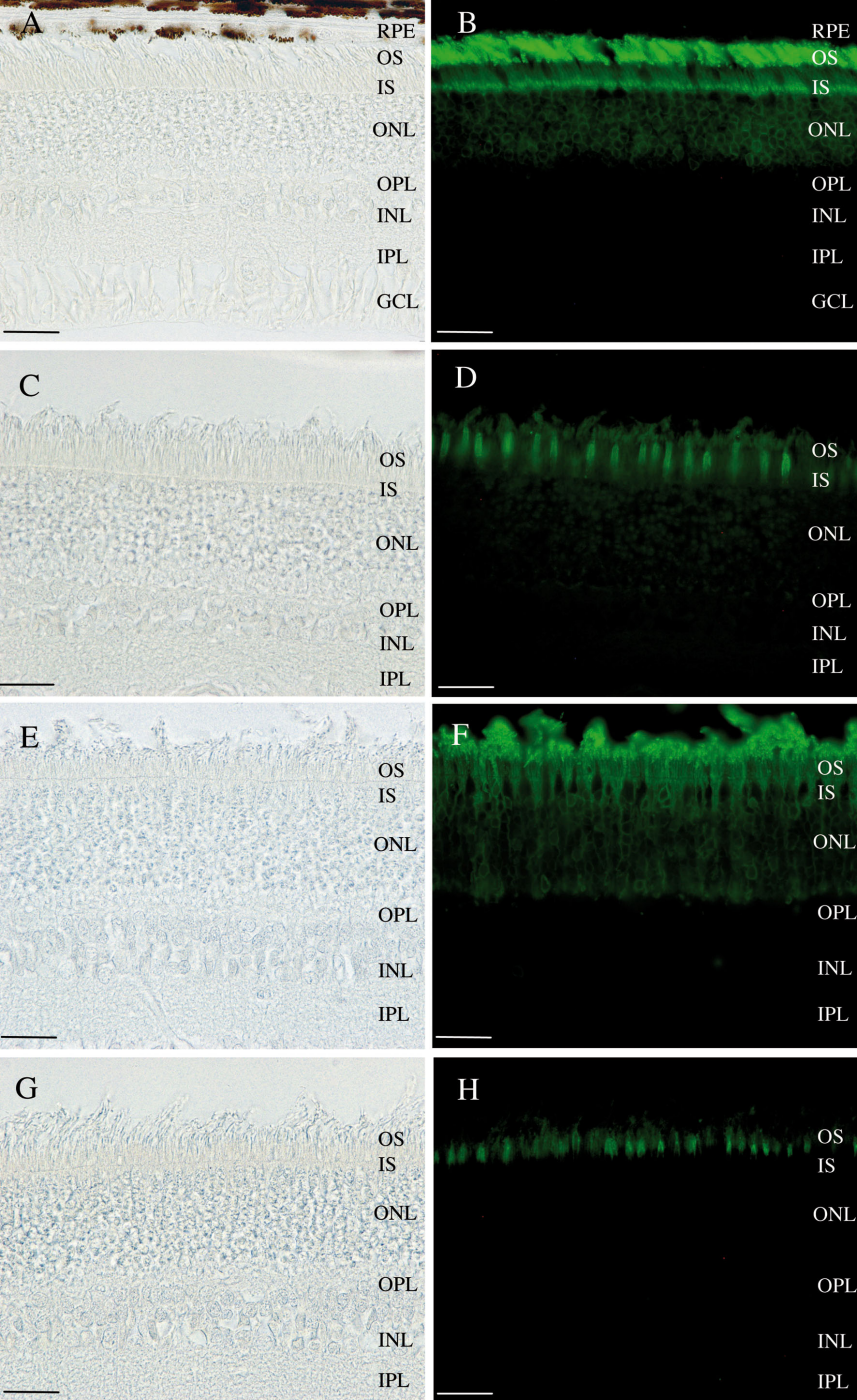
Light micrographs and immunofluorescent staining. **A,B,C,D:** Retina of the non affected dog NA2. **E,F,G,H:** Retina of the 2 months old affected dog A6. **B,F:** Rod labeling with anti-Rho4D2 antibody. **D,H:** Cone labeling with PNA-FITC lectin. These results show that both the rods and cones are still present at the age of 2 months in affected dogs. Abbreviations: retinal pigment epithelium (RPE); outer segments (OS); inner segments (IS); outer nuclear layer (ONL); outer plexiform layer (OPL); inner nuclear layer (INL); inner plexiform layer (IPL); ganglion cell layer (GCL). The scale bar represents 50 µm.

Immunofluorescence staining of the retina of A6 was performed, using the PNA-FITC lectin and the Rho4D2-FITC antibody. Distinct cone and rod labeling (Figure 6F,H) were observed, suggesting that rods and cones were still present at 2 months of age.

**Figure 7:**
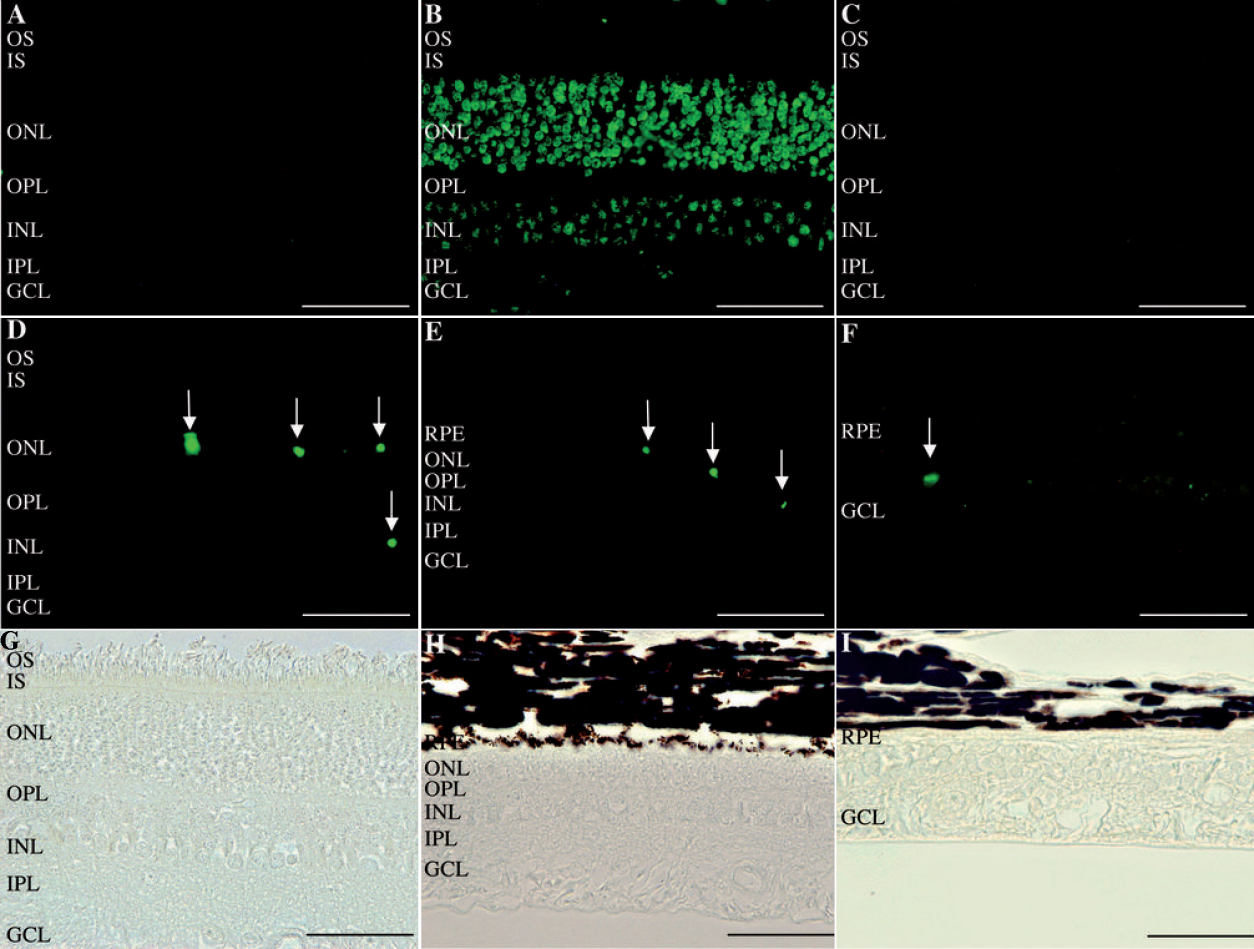
TUNEL assay on normal and affected dogs. **A:** Retina of the normal dog NA2 displayed no TUNEL-positive labeling. **B:** Retina of the affected dog A6 at the age of 2 months treated with DnaseI (positive control). **C:** Retina of the affected dog A6 at the age of 2 months treated with label solution only (negative control). TUNEL staining (**D,E,F**) and corresponding light micrographs (**G,H,I**) of the retinas of affected dogs. (**D,G**) retina of the affected dog A6 at the age of 2 months, (**E,H**) retina of the affected dog A1 at the age of 11 months and (**F,I**) retina of the affected dog A2 at the age of 28 months. TUNEL staining revealed apoptotic cells in all affected dogs, mostly in the outer nuclear layer, indicating apoptosis of photoreceptor cells. Abbreviations: retinal pigment epithelium (RPE); outer segments (OS); inner segments (IS); outer nuclear layer (ONL); outer plexiform layer (OPL); inner nuclear layer (INL); inner plexiform layer (IPL); ganglion cell layer (GCL). The scale bar represents 100 µm.

To test whether apoptotic cell death was the cause of retinal thinning of over time, we employed the TUNEL technique to stain sections from A6, A1, and A2 for apoptotic cells. Retina sections from A6, A1, and A2 were positive for TUNEL compared to normal retina. Most of the apoptotic nuclei were detected in the outer nuclear layer, with some nuclei detected in the inner nuclear layer and no signal in the ganglion cell layer (Figure 7).

## Discussion

In this study, *RPGRIP1*-deficient MLHD dogs were monitored clinically to define the optimal therapeutic window for retinal gene therapy. Analysis of the ERG findings showed that as early as 2 months of age, cone function was lost, while rod function was preserved. While cone function was undetectable at this time point, the cone photoreceptors themselves were still present in the retina at this early age. At 9 months of age, both cone and rod functions were undetectable. Interestingly, at this time point, assessment of each dog’s ability to avoid obstacles showed that functional vision is retained up to the age of 11 months. Both OCT and histopathology studies revealed a progressive thinning of the neuroretina over the first 2 years of age. TUNEL assay indicated that the apoptotic photoreceptor cell death was the mechanism of this thinning of the neuroretina.

In contrast to the study by Curtis and Barnett [7], which described what was thought to be a rod-cone dystrophy in the MLHD, and in accordance with the recent findings of Turney et al. [8], our results indicated that the *RPGRIP1*-deficient MLHD manifested a cone-rod dystrophy. Our ERG findings matched the Turney et al. [8] study. In their study, the ERG of a 6-week-old affected MLHD showed significant reduction of the 30 Hz flicker response.

Cone-rod dystrophies are rare. This is the first canine model of cone-rod dystrophy with a mutation that has been characterized [9]. A veterinary ophthalmological report from Kijas et al. [19] described a cone-rod dystrophy in pit bull terrier dogs; however, the causal gene was not identified. In this last model, the cone dysfunction was synchronous with the rod dysfunction, in contrast to what we have documented in the *RPGRIP1*-deficient model, where the rod responses appear to be relatively better preserved early in disease. Another cone-rod dystrophy was described in a standard wire haired dachshund, also displaying early functional changes of cones [20].

Although the ERG recordings were undetectable at the age of 9 months in several *RPGRIP1-*deficient dogs, these same affected dogs still retained some residual functional vision at this same age point. Functional vision persisted in at least one dog up to 14 months of age, at a time when the ERG was undetectable in this animal. This analysis suggests that evaluation both of functional vision using a behavioral test, as well as retinal function, using ERG, will have to be performed to evaluate the efficacy of any future treatment using this model. This is in sharp contrast to what we have observed in gene therapy treated *RPE65^−/−^* dogs, where rescue of retinal function was directly associated to an improvement of functional vision as assessed by behavioral studies [3].

**Figure 8:**
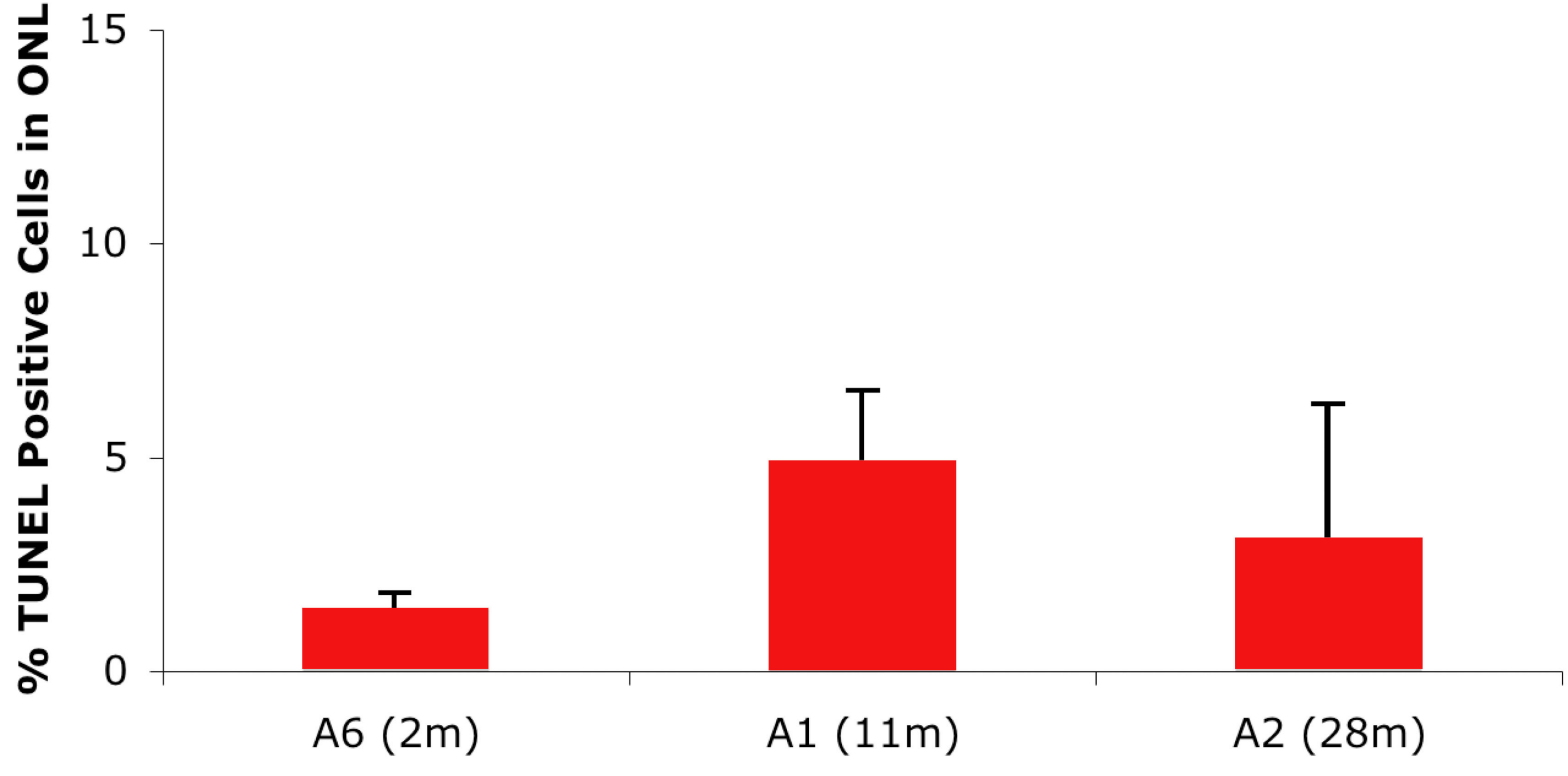
Quantification of TUNEL-positive cells in the outer nuclear layer of affected dogs. Apoptosis analysis was performed with fluorescent microscopy and a 40× objective. Terminal dUTP nick end labeling (TUNEL) positive cells stained with fluorescein were visualized and counted. TUNEL-positive cells in the outer nuclear layer (ONL) were quantified as the percent of the total number of cells in ONL per field. Data are the mean±standard error of the mean from 10 fields of view. The graph represents the average of 3 independent experiments. Months are abbreviated as m.

The progressive thinning of the ONL observed by OCT and on the retinal sections was due to apoptotic photoreceptor cell death. The photoreceptor cell death appears to progress in a similar rate over the first 2 years since the proportion of TUNEL-labeled photoreceptors remains consistent among the 3 different time points (2, 11, and 28 months; Figure 8). These results in the MLHD are different from those observed in the X-linked progressive retinal atrophy 2 (XLPRA2), which is characterized genetically by a frameshift mutation in exon 15 of the *RPGR* gene. In this model, a biphasic pattern of cell death was found, with an initial phase, from 4 to 12 weeks of age, and then a more gradual decrease after 12 weeks [21].

It was surprising to observe that for the 10-year-old *RPGRIP1*-deficient dog, not only had the outer retina disappeared but so had the inner retina. An analysis of hematoxylin- and eosin-counterstained sections from dogs between 2 months and 10 years of age did not reveal any inflammatory cells. To our knowledge, this phenomenon of complete neuroretinal degeneration has not been described in any other animal models of LCA or retinitis pigmentosa.

To date, the clinical literature on RPGRIP1-LCA patients clearly documents early and severe visual disturbances with nystagmus, abnormal visual acuity, nondetectable ERGs, and fundus features of retinal degeneration [22,23]. The close similarities between the clinical disease characteristics resulting from *RPGRIP1* gene mutations in humans and in the dog make this *RPGRIP1*-deficient MHLD a valuable model for the evaluation of gene therapy.

Recent success of AAV-mediated *RPGRIP1* gene transfer in a murine model of RPGRIP1-LCA is encouraging [24]. However, the evaluation of pre-clinical gene therapy in the eye of canine genetic models is far more desirable with respect to future clinical development; the canine eye is anatomically more similar to the human eye than the mouse counterpart. The dog ocular anatomic features are of similar proportion. Also, the surgical procedures for vector delivery and the amount of vector injected in the dog will be similar to what will be used in humans. In addition, dogs, which are relatively outbred compared to murine models, have greater immunological and biologic similarity to humans, which is vital and essential for determining the tolerance of vectors before clinical trials.

Recombinant AAV-mediated gene therapy has already been shown successful in the first canine model of LCA, the *RPE65^−/−^* dog. This *RPGRIP1*-deficient model is an attractive alternative LCA canine model in the sense that, in contrast to *RPE65*, it is not a rod-cone dystrophy but a cone-rod dystrophy and that the cells to target are not the RPE but the photoreceptors. A photoreceptor defect is more difficult to treat than a RPE defect, and a large animal model of such a defect has great intrinsic value.

In conclusion, we have characterized the kinetics of functional and structural changes that occur in *RPGRIP1*-deficient dogs. The results of this present study suggest a therapeutic strategy that consists of initiating gene therapy as early as possible after birth. Considering the small size of the MLHD dogs (120–200 g at birth) and our preliminary surgical data (unpublished results), accurate transvitreal subretinal injection is not possible in dogs younger than 2 months. Successful subretinal delivery of a therapeutic vector will be feasible by 2 months of age and may prevent or delay the loss of rod function. This is a valuable spontaneous animal model that represents a unique and important tool to assess the in vivo efficacy of photoreceptor targeted gene based therapeutic strategies for cone-rod dystrophies.

## Appendix 1. Behavioral tests.

Visual function was evaluated using behavioral testing based on the ability of each animal to avoid obstacles in normal light. The treated affected A2 avoided obstacles at the age of 8 months but consistently failed to avoid obstacles after the age of 11 months, seen in the movie in Appendix 1. To access the movie, click or select the words “Appendix 1.” This will initiate the download of a compressed (.mov) archive that contains the movie.
